# Knowledge, Attitudes, and Behavior Toward COVID-19 Among Jordanian Residents During the Quarantine Period of the COVID-19 Pandemic: A National Survey

**DOI:** 10.1017/dmp.2021.34

**Published:** 2021-02-16

**Authors:** Mahmoud Al-Hussami, Mamdouh El-Hneiti, Ayman Bani Salameh, Loai Abu Sharour, Rawan Al-Hussami

**Affiliations:** 1 The University of Jordan, School of Nursing, Amman, Jordan; 2 College of Nursing, Al-Zaytoonah University of Jordan, Amman, Jordan; 3 The University of Jordan, School of Medicine, Amman, Jordan

**Keywords:** communicable diseases, disease outbreaks, emergency preparedness, epidemiological monitoring

## Abstract

**Objectives:**

Coronavirus disease 2019 (COVID-19) is a communicable disease transmitted via respiratory droplet from 1 person to another caused by the severe acute respiratory syndrome coronavirus 2 (SARS-CoV-2). This study aims to investigate the knowledge, attitudes, and practice of Jordanian people toward COVID-19 during the COVID-19 pandemic. In addition, the paper explores the lack of perception and adherence to preventive measures toward COVID-19.

**Methods:**

A quantitative, cross-sectional, descriptive online survey was used to explore study variables. A convenience sample of individual who are of Jordanian nationality, were aged 18 years or older, understood the content of the questionnaire, and agreed to participate voluntarily was surveyed.

**Results:**

The average correct score of COVID-19 knowledge was 84.44% (12.66/15). In addition, knowledge scores significantly differed across demographic characteristics of participants. Moreover, 93.8% (1009) of the study sample had confidence that Jordan can win the battle against the COVID-19 virus. However, study participants acknowledged that they did not visit crowded places in recent days (91.6%), while 71.3% (767) wore masks when leaving home.

**Conclusions:**

The current study added a new knowledge that generally the Jordanian people during the quarantine period have a high knowledge and optimistic attitudes and practices toward COVID-19.

Coronavirus disease 2019 (COVID-19) is a communicable disease transmitted by means of respiratory droplet from 1 person to another caused by the severe acute respiratory syndrome coronavirus 2 (SARS-CoV-2).^1^ This infectious disease first appeared in Wuhan, the capital of China’s Hubei Province in December of 2019 then spread globally, resulting in the ongoing 2019-2020 coronavirus pandemic.^2^ The current COVID-19 epidemic has spread very quickly, and by April 6, 2020, the virus had reached almost all countries of the world, resulting in over 1.6 million confirmed cases and 97,179 deaths, with nearly all infections and deaths occurring in Europe.^3^

Common symptoms of COVID-19 include inflammation of the upper respiratory tract and symptoms similar to the flu, such as sneezing, coughing, sinus obstruction, and mucous secretions from the nose.^4^ After that, the temperature seems to rise to approximately 39 degrees or more during days 7-10, and then the person may feel a partial improvement until the end of the 2-wk period. While the majority of cases result in mild symptoms, some progress to acute injury to the lower respiratory tract and pneumonia. In addition to affecting the respiratory system, there is a high risk of death in elderly people and people of all ages who suffer from serious health conditions, such as heart disease, lung disease, and diabetes.^5^

Regarding the knowledge of the public toward COVID-19, across demographic groups, considerable numbers of American people reported having heard “a lot” about the coronavirus, including substantial shares across gender, education levels, and health status. Adults who are aged 50 and older are somewhat more likely than younger adults to have heard or read “a lot” about the coronavirus compared with other age groups, with approximately half of adults under age 50 confirming that they have heard or read “a lot,” and this share increased to nearly two-thirds among adults ages 50 and older. In addition, approximately half of adults with less than a 4-y college degree say that they have heard “a lot,” and this percentage increases to 66% among those with a college degree or higher. These findings related to age and education from previous surveys demonstrate that older adults and those with higher levels of education are more likely to pay closer attention to health news.^6^ A deeper understanding of knowledge-based awareness and behavioral adaptation is, however, increasingly recommended to contain COVID-19.^7^

Regarding people’s attitudes toward coronavirus, the Institut de Publique Sondage d’Opinion Secteur (Ipsos) (2020)^8^ have done a national survey and reported that, in 13 of the 15 countries, majorities raised concern for those who are weak and vulnerable at the top of a list of 10 options when they asked what best describes their feeling today. People in Brazil (70%), Spain and the United Kingdom (66%), Mexico (61%), and Canada, France, and Italy (60%) are mostly likely to express concern for others, while those in Japan (23%) and China (30%) are least worried. In addition, a majority (53%) of people reported that this pandemic is more likely to bring them closer to their family and friends. This finding was strongest in Asian countries of India (72%), Vietnam (70%), and China (67%), while those in Japan (19%), South Korea (32%), and Germany (41%) are least likely to agree with this. Studying people’s attitudes of the crisis COVID-19 is important to predict the behavior of the people in their own environments.^9^

The coronavirus is highly infectious and has a high fatality rate, with more than 15 thousand deaths in Italy and more than 12 thousand deaths in Spain.^3^ People all over the world should adhere to control measures as the battle against this epidemic is continuing. These control measures, however, are influenced by people’ knowledge, attitudes, and practices (KAP) according to KAP theory^9^; therefore, exploring these variables could prevent the spread of the disease.

The knowledge, attitudes, and behavior of Jordanian people toward the COVID-19 and their willingness to protect themselves during its outbreak have not yet been investigated. This study aims to investigate the knowledge, attitudes, and behavior of Jordanian people toward COVID-19 during the COVID-19 pandemic. In addition, this study explores the lack of perception and adherence to preventive measures toward COVID-19.

## Methods

### Design

A quantitative, cross-sectional, descriptive design was used to explore knowledge, attitudes, and practices toward COVID-19 among Jordanian adult. This cross-sectional survey was conducted using an online survey from March 20 to the middle of April 2020, the 4 wk immediately after the lockdown of Jordan. This method was chosen, as it was believed to be the most appropriate technique during the COVID-19 crisis and quarantine being imposed in Jordan.

### Setting, Sample, and Sample Size

A convenience sample of individuals who are of Jordanian nationality, were aged 18 years or older, understood the content of the questionnaire, and agreed to participate in the study were instructed to complete the questionnaire by means of clicking the link through Facebook or WhatsApp. To meet overall study goals, a power analysis was conducted using G*power program^10^ to determine the required sample size, a proportion chi-squared (*χ* ^2^) analysis with a medium effect size of 0.30, a statistical power 0.80, and a significant alpha 0.05. The calculated minimal sample size needed for the study was 1050; however, 1078 individuals completed the survey.

### Ethical Considerations

Institutional Review Board (IRB) approval from Al-Zaytoonah University of Jordan approved the study protocol and procedures of informed consent before the formal survey. Participants instructed to read the informed consent carefully and had to answer a yes-no question at the end of the consent form if they are willing to participate voluntarily. The consent form contains detailed descriptions on the purpose of the study, its benefits, risks, and procedures, and indicated that each subject’s enrollment in the research project is completely voluntarily. After confirmation of the question, the participant was directed to complete the self-report questionnaire.

### Measurement

The questionnaire consisted of 5 parts: sociodemographic characteristics, knowledge, attitude, and practices toward COVID-19, and compliance of hand washing. The first section of the questionnaire concerned demographic variables, including age, gender, marital status, education, occupation, and place of current residence. The second section of the questionnaire concerned COVID-19 knowledge, adapted from the Zhong et al. (2020)^11^ KAPs questionnaire and based on the WHO guidelines. The questionnaire consisted of 15 questions divided on 3 themes: clinical presentations were 4 questions, transmission routes were 3 questions, and prevention and control of COVID-19 were 8 questions. The answer options per question were true/false and I do not know. The correct answer was given 1 point, while incorrect/unknown answers were given zero points. This questionnaire has been reliable when used in previous settings,^11^ and the Cronbach’s alpha coefficient was 0.82 in the current study.

The third section, attitudes toward COVID-19, measured by 2 questions, concerned agreement that COVID-19 will finally be successfully controlled and the confidence that Jordan can win the battle against the COVID-19 virus. The answer options per question were agree/disagree and I do not know. The fourth section, the assessment of participants’ practices, included 3 behaviors: gone to any crowded places, worn a mask when leaving home, worn gloves when leaving home. The answer options per question were yes/no and I do not know. The fifth section, self-reported hand washing subscale, was developed by the authors and asks respondents to rate the percentage of their compliance to hand washing (from 10 to 100%).

In addition, participants were asked about hand washing performance in 6 different circumstances (eg, before starting to provide the service to oneself or others, when to stop the service promptly due to a phone call, when buying directly from a store, after touching surrounding surfaces, after removing gloves, and before touching your nose, mouth, eyes, or any other area of your face) (Table 1). Also, it asks about behavior related to controlling COVID-9 infection through the hands cleaning process. Hand washing practice were sought by a 10-point percentage scale, with 10% indicating minimal hand washing and 100% indicating maximal hand washing.

**Table 1.** Study items

**Knowledge**
1. The main clinical symptoms of COVID-19 are fever, fatigue, dry cough, and myalgia
2. Unlike the common cold, stuffy nose, runny nose, and sneezing are less common in persons infected with the COVID-19 virus.
3. There currently is no effective cure for COVID-2019, but early symptomatic and supportive treatment can help most patients recover from the infection.
4. Not all persons with COVID-2019 will develop to severe cases. Only those who are elderly, have chronic illnesses, and are obese are more likely to be severe cases.
5. Eating or contacting wild animals would result in the infection by the COVID-19 virus.
6. Persons with COVID-2019 cannot infect the virus to others when a fever is not present.
7. The COVID-19 virus spreads by means of respiratory droplets of infected individuals.
8. Ordinary residents can wear general medical masks to prevent the infection by the COVID-19 virus.
9. It is not necessary for children and young adults to take measures to prevent the infection by the COVID-19 virus.
10. I am not concerned about the harm to health I might have from Covid-19 infection.
11. I think that the scientific evidence about the Covid-19 virus that is harmful to health is overrated.
12.To prevent the infection by COVID-19, individuals should avoid going to crowded places such as train stations and avoid taking public transportations.
13. Isolation and treatment of people who are infected with the COVID-19 virus are effective ways to reduce the spread of the virus.
14. People who have contact with someone infected with the COVID-19 virus should be immediately isolated in a proper place.
15. In general, the occupation period for COVID-19 virus is 14 days.
**Attitudes**
1. Do you agree that COVID-19 will finally be successfully controlled?
2. Do you have confidence that Jordan can win the battle against the COVID-19 virus?
**Practices**
1. In recent days, have you gone to any crowded place?
2. In recent days, have you worn a mask when leaving home?
3. In recent days, have you worn gloves when leaving home?
**Individual Compliance to Hand Washing**
1. Wash hands before starting to provide the service to oneself or others.
2. Wash hands when you have to stop the service promptly (due to a phone call).
3. Wash hands when buying directly from a store.
4. Wash hands after touching the surfaces around.
5. Wash hands after removing gloves.
6. Wash hands before touching your nose, mouth, eyes, or any other area of your face.

Moreover, permission to use the questionnaire was granted by Professor Yi Li from Huazhong University of Science & Technology, Wuhan, China. In addition, the questionnaire was translated into the Arabic language, and WHO guidelines were followed for translation using the following steps: forward translation, expert panel back-translation, pretesting, and cognitive interviewing until reaching the final version.

### Data Collection Procedure

This study used an electronic survey. Confidentiality was ensured through designing the electronic survey with anonymity features in which no identifying information of the subjects was included. Furthermore, retrieved questionnaires were stored on a password-protected on-site file server.

### Statistical Analysis

The Statistical Package for Social Sciences version 25^12^ used for data entry and analysis. Descriptive statistics of frequencies, means, range, and standard deviations calculated to describe subjects’ demographic data. Mean scores and standard deviation around the mean calculated for the study variables (ie, KAPs). One-way analysis of variance (ANOVA) and independent χ^2^ tests were used to measure the differences between the means or cross-tabulate of the study variables as appropriate.

## Results

Demographically, the study participants (*n* = 1076) were 59.8% (643) female and 40.2% (433) male. The mean age of the participants was 34.83 ± 10.50 y, ranging from 18 to 70 y. Of the participants, 25% were 18-27 y old, with a median age of 34 y; 75% ranged from 40 to 70 y.

The majority (64.1%; *n* = 690) of the study participants were married, and 392 were female. Moreover, 370 of the sample were single, 65% (240) of them were female, while 16 participants were divorced. In addition, 32.1% of the participants earned less than 500 Jordanian Dinars a month, most participants 928 (86%) were university graduates, and 430 (20%) were Amman residents. Other demographic characteristics are shown in Table 2.

**Table 2.** Demographic features of participants

Demographic variable	No.	%
Age (y)		
≤ 29	364	33.8
30-49	590	54.8
≥50	122	11.3
Gender		
Male	433	40.2
Female	643	59.8
Marital status		
Single	370	34.4
Married	690	64.1
Divorced/widow	16	1.5
Monthly income (JD)		
Less than 500	345	32.1
500-1000	426	39.6
More than 1000	305	28.3
Level of education		
Some schooling	16	1.5
High school	132	12.3
University graduate	928	86.2

The average correct score of COVID-19 knowledge was 84.44% (12.66/15), ranging from 59.5% to 98.5% (8.92-14.77/15). Twenty-five percent (Q1) of the correct answers were 73.9%, while the median score is 87.3%, and Q3 was 97.6%. The highest correct answer (98.5%) was observed for the item, “*Isolation and treatment of people who are infected with the COVID-19 virus are effective ways to reduce the spread of the virus*.” Whereas the lowest correct answer (59.5%) was noted when the students answered the item “*Eating or contacting wild animals would result in the infection by the COVID-19 virus*” (Table 3). Moreover, Knowledge scores significantly differed across demographic characteristics of participants. The results showed that there are significant differences between age, marital status, monthly income, level of education, profession, and residency governorates (F = 3.90; *P* = 0.02; F = 3.26; *P* = 0.03; F = 5.41; *P* = 0.005; F = 16.13; *P* = 0.000; F = 4.98; *P* = 0.000; F = 2.23; *P* = 0.01, respectively) (Table 4).

**Table 3.** Knowledge toward COVID-19

Item #	No. of correct answers (%)	No. of false answers (%)	No. do not know (%)
1	1015 (94.3%)	27 (2.5%)	34 (3.2%)
2	795 (73.9%)	174 (16.2%)	107(9.9%)
3	939 (87.3%)	66 (6.1%)	71 (6.6%)
4	682 (63.4%)	305 (28.3%)	89 (8.3%)
5	640 (59.5%)	211 (19.6%)	225 (20.9%)
6	923 (85.8%)	45 (4.2%)	108 (10.0%)
7	903 (83.9%)	70 (6.5%)	103 (9.6%)
8	669 (62.2%)	339 (31.5%)	68 (6.3%)
9	1050 (97.6%)	10 (.9%)	16 (1.5%)
10	979 (91.0%)	67 (6.2%)	30 (2.8%)
11	862 (80.1%)	118 (11.0%)	96 (8.9%)
12	1058 (98.3%)	6 (.6%)	12 (1.1%)
13	1060 (98.5%)	8 (.7%)	8 (.7%)
14	1054 (98.0%)	10 (.9%)	12 (1.1%)
15	998 (92.8%)	52 (4.8%)	26 (2.4%)
Total	Mean and SD of knowledge score = 84.40 (13.79), range (59.50-98.50)		

**Table 4.** Knowledge score of COVID-19 by demographic characteristics of participants

Characteristics	No. of participants (%)	Knowledge score/10(SD)	F	*P*
Age (y)				
Less than 29	364 (33.8)	8.76 (1.68)	3.90	0.020
30-49	590 (54.8)	9.05 (1.44)		
Above 50	122 (11.3)	8.91 (1.64)		0.020
Gender				
Male	433 (40.2)	8.85 (1.56)	1.47	0.140
Female	643 (59.8)	9.00 (1.43)		
Marital status				
Married	690 (34.4)	8.90 (1.43)	3.26	0.03
Single	370 (64.1)	9.00 (1.65)		
Divorced/widow	16 (1.5)	8.00 (3.24)		
Monthly income (JD)				
Less than 500	345 (32.1)	8.70 (1.90)	5.41	0.005
500-1000	426 (39.6)	8.90 (1.28)		
More than 1000	305 (28.3)	9.10 (1.44)		
Level of education				
Some schooling	16 (1.5)	6.20 (3.29)	16.13	0.000
High school	132 (12.3)	8.60 (1.98)		
University	928 (86.2)	9.80 (1.40)		
Profession				
Faculty members	58 (5.4%)	9.60 (1.18)	4.98	0.000
School teachers	90 (8.4%)	9.00 (1.48)		
Health practitioners	243 (22.6%)	9.20 (1.01)		
Engineers	67 (6.2%)	9.20 (1.19)		
Govern. employees	94 (8.7%)	8.70 (1.95)		
Army and police	50 (4.6%)	8.80 (1.65)		
Blue collar workers	149 (13.8%)	8.80 (1.58)		
College students	97 (9%)	8.40 (2.02)		
Retirements	63 (5.9%)	9.20 (1.24)		
Unemployed	165 (15.3%)	8.60 (1.68)		
Governorate				
Amman	430 (40.0%)	8.90 (1.47)	2.23	.01
Al-Balqa	188 (17.5%)	9.20 (1.65)		
Zarqa	68 (6.3%)	8.40 (1.41)		
Jarash	45 (4.2%)	8.80 (1.66)		
Ajloun	40 (3.7%)	9.00 (1.20)		
Irbid	62 (5.8%)	8.80 (1.41)		
Al-Mafraq	34 (3.2%)	8.70 (1.46)		
Madaba	28 (2.6%)	8.20 (1.69)		
Al-Karak	47 (4.4%)	9.00 (1.59)		
Al-Tafila	40 (3.7%)	8.80 (1.43)		
Maan	42 (3.9%)	8.60 (2.35)		
Aqaba	52 (4.8%)	9.30 (1.32)		

Note: ** *P* < 0.01, * *P* < 0.05.

It is noted that 78% of the Jordanian people who filled out the survey online during the 4-wk quarantine agreed that the government will be successful in controlling the pandemic COVID-19, while 93.8% (1009) of the study sample had confidence that Jordan can win the battle against the COVID-19 virus. Moreover, most study participants acknowledged that they did not visit crowded places in recent days (91.6%), while 71.3% (767) worn masks and 69.1% (743) worn gloves when leaving homes (Table 5). In addition, when demographic characteristics were cross-tabulated with attitude scores, the results with the first item, successfully controlled COVID-19, showed that there are significant differences between participants professions, and residency governorates (χ^2^ = 37.3; *P* = 0.01; χ^2^ = 47.3; *P* = 0.001, respectively). The attitude of the participants toward winning the battle against COVID-19 results significantly differed across the categories level of education, profession, and residence of governorate (χ^2^ = 32.8; *P* = 0.000; χ^2^ = 81.3; *P* = 0.000; χ^2^ = 44.7; *P* = 0.003, respectively) (Table 6).

**Table 5.** Attitude and practices toward COVID-19

Variable	Item no.	No. agree (%)	No. disagree (%)	No. I do not know (%)
Attitudes	1	843 (78.3%)	115 (10.7%)	118 (11.0%)
	2	1009 (93.8%)	14 (1.3%)	14 (4.9%)
Practices	Item no.	No. yes (%)	No. no (%)	No. I do not know (%)
	1	86 (8.0%)	986 (91.6%)	4 (.4%)
	2	767 (71.3%)	303 (28.2%)	6 (.6%)
	3	743 (69.1%)	327 (30.4%)	6 (.6%)

**Table 6.** Attitude toward COVID-19 by demographic characteristics

Variables	Attitudes of Jordanian people toward COVID-19 (N)									
	Successfully controlled COVID-19					Winning the battle against COVID-19				
	Agree	Disagree	Don’t know	*χ* ^2^	P	Yes	No	Don’t know	*χ* ^2^	P
Gender										
Female	494	73	76	2.19	.331	604	35	4	6.53	.038
Male	349	42	42			405	18	10		
Age (y)										
Less than 29	288	42	34	2.30	.681	341	19	4	4.91	.296
30-49	463	59	68			554	30	6		
Above 50	92	14	16			114	4	4		
Marital status										
Single	287	51	32	9.28	.050	345	21	4	3.30	.508
Divorced	14	1	2			14	2	1		
Married	542	64	84			650	30	10		
Household income (JD)										
< 500 a month	271	37	37	3.54	.47	322	19	4	.45	.97
500-1000 a month	327	44	55			400	20	6		
>1000 a month	245	34	26			287	14	4		
Level of education										
Less than 7 grades	10	2	4	7.18	.304	14	1	2	32.8	.000
High school	102	18	12			120	7	4		
University graduate	731	95	102			875	45	8		
Profession										
Faculty members	44	9	5	37.3	.011	57	1	1	81.3	.000
Unemployment	135	8	22			155	10	2		
School teacher	76	4	10			89	1	3		
Health practitioner	182	35	26			227	13	3		
Engineer	54	7	6			64	3	1		
Clerical Employee	61	18	15			78	6	7		
Military & police	42	2	6			48	1	2		
Blue collar workers	121	15	13			140	9	1		
College student	71	14	12			90	7	1		
Retirement	57	3	3			61	2	2		
Governorate										
Amman	327	61	42	47.3	.001	401	20	5	44.7	.003
Tafila	35	1	4			40	1	1		
Maan	27	6	9			39	3	1		
Aqaba	43	6	9			51	1	1		
Balqa	159	12	17			184	2	2		
Zarqa	47	8	13			62	6	1		
Jarash	38	3	4			44	1	1		
Ajloun	32	3	5			38	1	1		
Irbid	49	1	12			56	6	2		
Mafraq	24	8	2			31	2	1		
Madaba	25	3	1			26	2	2		
Karak	37	3	7			37	9	1		

Note: ** *P* < 0.01, * *P* < 0.05.

With regard to the practice of the study sample toward going to a crowded place, it was found only gender from the demographics was statistically significant (χ^2^ = 18.3; *P* = 0.000). Also, results of the second item, wearing a mask to protect self from COVID-19 virus, showed that gender, age, household, profession, and residence of governorate were statistically significant (χ^2^ = 6.12; *P* = 0.04; χ^2^ = 15.3; *P* = 0.004; χ^2^ = 28.6; *P* = 0.000; χ^2^ = 78.4; *P* = 0.000; χ^2^ = 66.3; *P* = 0.000, respectively) (Table 7). The average score of self-reported hand washing compliance among Jordanian people was 83.50% (SD = 21.20). The highest compliance rate (mean = 87.88%; SD = 21.85) was observed for the item “*Wash hands when buying directly from a store*,” whereas the lowest compliance rate (mean = 73.0%; SD = 25.41) was noted for the item “*Wash hands when you have to stop the service promptly (due to a phone call)*” (Table 8).

**Table 7.** Practices toward COVID-19 by demographic characteristics

Variables	Practices of Jordanian people toward COVID-19 (N)									
	Going to a crowded place					Wearing a mask to protect self from COVID-19 virus				
	Yes	No	Don’t know	*χ* ^2^	P	Yes	No	Don’t know	*χ* ^2^	P
Gender										
Female	34	605	4	18.3	.000	467	170	6	6.12	.047
Male	52	381	1			300	133	1		
Age (y)										
Less than 29	25	337	2	2.56	.634	233	129	2	15.3	.004
30-49	48	540	2			441	145	4		
Above 50	13	109	1			93	29	1		
Marital status										
Single	36	322	2	5.84	.211	255	115	1	7.18	.127
Divorced	3	13	2			14	2	1		
Married	47	641	1			49	186	6		
Household income (JD)										
<500 a month	31	312	2	2.65	.611	224	117	4	28.6	.000
500-1000 a month	30	394	1			292	132	2		
>1000 a month	25	280	2			251	54	1		
Level of education										
Less than 7 grades	4	12	1	11.8	.066	12	4	1	5.65	.46
High school	16	116	1			92	38	2		
University graduate	66	858	4			663	261	1		
Profession										
Faculty members	6	52	1	27.3	0.12	40	18	1	78.4	.000
Unemployment	10	155	2			95	64	2		
School teacher	3	87	1			69	21	1		
Health practitioner	24	217	2			196	47	1		
Engineer	5	62	1			38	29	1		
Clerical Employee	7	87	2			78	16	2		
Military & police	8	42	2			35	15	2		
Blue collar workers	16	133	2			104	45	1		
College student	2	93	2			62	35	1		
Retirement	5	58	1			50	13	2		
Governorate										
Amman	47	381	2	32.3	.072	318	110	1	66.3	.000
Tafila	4	36	1			26	13	2		
Maan	6	36	1			27	15	1		
Aqaba	4	48	1			41	11	1		
Balqa	8	180	1			138	50	2		
Zarqa	2	66	1			26	42	1		
Jarash	4	41	1			31	12	2		
Ajloun	3	36	1			31	9	1		
Irbid	4	58	1			47	15	2		
Mafraq	1	33	1			23	11	1		
Madaba	3	25	1			23	5	2		
Karak	1	46	1			36	10	1		

Note: ** *P* < 0.01, * *P* < 0.05.

**Table 8.** Compliance of individuals from 100% toward hand washing to control COVID-19

Item no.	10 (%)	20 (%)	30 (%)	40 (%)	50(%)	60(%)	70 (%)	80(%)	90(%)	100(%)
1	20(1.9)	8(.7)	8(.7)	13(1.2)	48(4.5)	31(2.9)	82(7.6)	160(14.9)	186(17.3)	520(48.3)
2	65(6.0)	15(1.4)	23(2.1)	24(2.2)	122(11.3%)	88(8.2)	130(12.1)	200(18.6)	143(13.3)	266(24.7)
3	32(3)	14(1.3)	12(1.1)	3(.3)	32(3)	46(4.3)	66(6.1)	71(6.6)	118(11.0)	682(63.4)
4	28(2.6)	8(.7)	10(.9)	34(3.2)	40(3.7)	43(4)	61(5.7)	97(9.0)	164(15.2)	591(54.9)
5	48(4.5)	8(.7%)	17(1.6)	16(1.5)	38(3.5)	29(2.7)	57(5.3)	95(8.8)	121(11.2)	647(60.1)
6	40(3.7)	14(1.3)	28(2.6)	16(1.5)	42(3.9)	64(5.9)	100(9.3)	128(11.9)	138(12.8)	506(47.0)

Pearson product-moment correlation was conducted to assess the relationship of the knowledge score with attitudes, practices, and the compliance to hand washing. As shown in Table 9, there was a significant positive relationship between knowledge score and attitude (r = 0.216; *P* < 0.001), knowledge score with practices (r = 0.128; *P* < 0.001), and knowledge score with score compliance to hand washing (r = 0.177; *P* < 0.001). However, the knowledge score was not significantly correlated with age of the study participants (r = 0.047; *P* > 0.05).

**Table 9.** Correlation between knowledge score, attitude, practices, compliance to hand washing, and participants’ age

Variable	Knowledge	Attitude	Practices	Hand Washing	Age
Knowledge	1				
Attitude	0.216**	1			
Practices	0.128**	0.097**	1		
Hand washing compliance	0.177**	0.132**	0.096**	1	
Age	0.047	-0.066*	0.085**	0.057	1

Note: ** *P* < 0.01, * *P* < 0.05.

## Discussion

The current study aimed to investigate the knowledge, attitudes, and behavior of Jordanian people toward COVID-19 during the COVID-19 pandemic. This national study found a high knowledge of COVID-19 among the sample, with the highest level of knowledge relating to the effective way to reduce the spread of virus by isolation and treatment of people who are infected with COVID-19. This finding was similar to another study conducted in China.^11^ This high knowledge among the current study population could be explained by the different resources of information about the disease from media news, social media, official government websites as well as family and friends. Another explanation could be related the characteristics of the sample with the majority holding a bachelor degree or higher. Additionally, those participants who reported a high knowledge of the disease were significantly more educated and were employed. It is useful, therefore, for public health policy-makers and health workers to recognize that those who are not employed or well educated will need more information about the disease.

Regarding the attitudes of the sample population, the majority of the participants reported positive perceptions about the government measures to control the COVID-19 pandemic and reported that Jordan can win the battle against the COVID-19 virus. Those who graduated high school or less, however, were more likely to report negative attitudes compared with those who hold a bachelor degree or higher. The Jordanian government recently has imposed regulations to reduce the spread of the virus by, for instance, on March 20, 2020, the Government of Jordan announced that the nationwide curfew for 4 wk in which this curfew prohibits the movement of people and closes most shops. Additionally, all travelers who arrived in Jordan on March 16 were immediately quarantined for 14 days in hotels in Amman and at the Dead Sea. These governmental measures could explain the positive attitudes of the participants toward COVID-19.

Generally, the study participants reported positive practices during the quarantine period of the COVID-19 pandemic. The majority said that they did not visit crowded places in recent days, and they wore masks and gloves when leaving homes. These practices could be contributed to the governmental preventive measures that have been enforced in Jordan, such as prohibition on all movement between governates, citizens may not leave home except in extreme circumstances, and prohibition on gatherings of more than 10 people. Nowadays, the increasing occurrence rate and mortality rate of the COVID-19 across the world and in particular in Middle East countries (eg, Iraq, Iran, and Lebanon) could raise Jordanians’ awareness about the disease and its prevention methods. The updated information about the spread of COVID-19 worldwide by national news and international organizations, such as WHO^3^ and CDC,^4^ have increased the knowledge of the people to comply with positive practices. Despite these positive results, the current study found significant differences between gender and residents went to crowded places in which males were more likely to go to crowded places compared with the females. This could be explained by the current practices during the quarantine period in Jordan in which men usually buy goods from stores, whereas females remain at home to take care of children. This could make the males at higher risk to obtain the disease than females.^13-15^ As of May 5, Jordan had 465 confirmed COVID-19 cases: 40% were females, 367 recovered, and 9 died.

With regard to hand washing practices, the majority of the participants reported positive practices toward hand washing. The highest compliance was related to washing hands when buying directly from a store, whereas the lowest compliance rate was about washing hands when you have to stop the service promptly (due to a phone call). The WHO^3^ recommended that hand washing for 20 s with soap and water or with alcohol-based hand rub could reduce the risk of getting the virus. However, people take their phones everywhere, and they are constantly touching them; if they have been out and their phone is contaminated, this could be a source of COVID-19 infections, given that this virus is so contagious and it can survive on surfaces for 2 or 3 d. Public policy-makers and health professionals in Jordan need to emphasis on the importance of hand washing and in particular when people have to stop the service, for instance to answer a phone call.

In this study, the findings indicated that there was a strong positive association of people’s higher knowledge scores with higher positive attitudes, hand washing compliance, and safe practices toward the COVID-19 epidemic. These findings were in line with previous studies carried out by Zhou et al.^16^ and Alzoubi et al.^17^ Moreover, our finding of the age factor associated with KAP toward COVID-19 are generally consistent with previous studies, such as those by Giao et al.^18^ and Zhong et al.^11^ The study results clearly indicate the importance of improving people’s COVID-19 knowledge by means of health education, which may also result in improvements in their attitudes and practices toward COVID-19.

The current study provided an in-depth view of Jordanian behavior toward COVID-19. In addition, it added benefit for policy makers to see how things work during the quarantine period. Moreover, the study participants were recruited from different governates in Jordan, which could increase the generalizability of the current study findings. In addition, the current study recruited a large and representative sample.

## Limitations

It is important to acknowledge the limitations of the current study. For instance, personal bias in the self-reporting method may have affected the results of this study. Indeed, an issue associated with a self-reporting method is that respondents may not answer truthfully due to an inability to recall or a desire to exaggerate issues. Nevertheless, it is important to consider participants’ responses regarding COVID-19 as a starting point for further research. In addition, this approach was effective in enabling the study to meet its aim and objectives. It allows participants to cooperate with the research by completing the questionnaires in their own time. It was not possible to conduct a random sampling method to recruit the participants, as the researcher has limited resources to access all the study population; therefore, a convenience sampling technique was used for the current study.

## Conclusions

The current study added the new knowledge that, generally, the Jordanian people during the quarantine period have a high knowledge and optimistic attitudes and practices toward COVID-19. This suggests that health education programs and governmental preventive measures to improve the COVID-19 knowledge and practices are effective. However, unemployed men are at higher risk to contract COVID-19 compared with other groups. Thus, health-care employers and policy-makers need to take into consideration that some population groups, including those who are not employed or well educated would need more information about the disease. Further research studies using observational methods (for instance) need to be conducted to confirm this investigation’s findings.
